# Identification of Proteases and Protease Inhibitors in Seeds of the Recalcitrant Forest Tree Species *Quercus ilex*

**DOI:** 10.3389/fpls.2022.907042

**Published:** 2022-06-27

**Authors:** Monica Escandón, Ezequiel D. Bigatton, Victor M. Guerrero-Sánchez, Tamara Hernández-Lao, Maria-Dolores Rey, Jesus V. Jorrín-Novo, Maria Angeles Castillejo

**Affiliations:** ^1^Agroforestry and Plant Biochemistry, Proteomics and Systems Biology, Department of Biochemistry and Molecular Biology, University of Córdoba, Córdoba, Spain; ^2^Agricultural Microbiology, Faculty of Agricultural Science, National University of Córdoba, CONICET, Córdoba, Argentina

**Keywords:** protease, protease inhibitors, nonorthodox seeds, germination, *Quercus ilex*, proteomics, protease activity

## Abstract

Proteases and protease inhibitors have been identified in the recalcitrant species *Quercus ilex* using *in silico* and wet methods, with focus on those present in seeds during germination. *In silico* analyses showed that the *Q. ilex* transcriptome database contained 2,240 and 97 transcripts annotated as proteases and protease inhibitors, respectively. They belonged to the different families according to MEROPS,^1^ being the serine and metallo ones the most represented. The data were compared with those previously reported for other *Quercus* species, including *Q. suber, Q. lobata,* and *Q. robur*. Changes in proteases and protease inhibitors alongside seed germination in cotyledon and embryo axis tissues were assessed using proteomics and *in vitro* and in gel activity assays. Shotgun (LC–MSMS) analysis of embryo axes and cotyledons in nonviable (NV), mature (T1) and germinated (T3) seeds allowed the identification of 177 proteases and 12 protease inhibitors, mostly represented by serine and metallo types. Total protease activity, as determined by *in vitro* assays using azocasein as substrate, was higher in cotyledons than in embryo axes. There were not differences in activity among cotyledon samples, while embryo axis peaked at germinated T4 stage. Gel assays revealed the presence of protease activities in at least 10 resolved bands, in the *Mr* range of 60–260 kDa, being some of them common to cotyledons and embryo axes in either nonviable, mature, and germinated seeds. Bands showing quantitative or qualitative changes upon germination were observed in embryo axes but not in cotyledons at *Mr* values of 60–140 kDa. Proteomics shotgun analysis of the 10 bands with protease activity supported the results obtained in the overall proteome analysis, with 227 proteases and 3 protease inhibitors identified mostly represented by the serine, cysteine, and metallo families. The combined use of shotgun proteomics and protease activity measurements allowed the identification of tissue-specific (e.g., cysteine protease inhibitors in embryo axes of mature acorns) and stage-specific proteins (e.g., those associated with mobilization of storage proteins accumulated in T3 stage). Those proteins showing differences between nonviable and viable seeds could be related to viability, and those variables between mature and germinated could be associated with the germination process. These differences are observed mostly in embryo axes but not in cotyledons. Among them, those implicated in mobilization of reserve proteins, such as the cathepsin H cysteine protease and Clp proteases, and also the large number of subunits of the CNS and 26S proteasome complex differentially identified in embryos of the several stages suggests that protein degradation *via* CNS/26S plays a major role early in germination. Conversely, aspartic proteases such as nepenthesins were exclusively identified in NV seeds, so their presence could be used as indicator of nonviability.

## Introduction

Germination is a complex process by which a seed embryo develops into a seedling. It is also a critical stage for plant development, survival, propagation and reproduction. Seeds can be classified as orthodox and nonorthodox (recalcitrant) according mainly to their tolerance to dehydration (Berjak and Pammenter, 2008). Orthodox seeds generally retain viability and germinability even after storage over long periods under suitable dry, cool conditions. On the other hand, nonorthodox seeds are damaged by, and cannot survive, dehydration (Roberts, 1973; Wyse and Dickie, 2017). *Quercus* species belong to the latter, recalcitrant group, so they must be shed and germinate immediately after maturation, when their moisture content is still relatively high (Connor and Sowa, 2002; Pasquini et al., 2012; Joët et al., 2013; Sghaier-Hammami et al., 2016). Therefore, after harvesting and processing recalcitrant seeds, storing them over long periods in unsuitable conditions can cause loss of their viability and pose serious difficulties to conservation and propagation of the species (Pasquini et al., 2011, 2012).

Seed germination has been widely studied in orthodox seeds. The process involves major changes including reprogramming of gene expression under hormonal control (Angelovici et al., 2010; Wang et al., 2015). By contrast, available knowledge of germination on the nonorthodox seeds such as those of *Q. ilex* is limited (Pammenter and Berjak, 2014) despite the fact that the storage, desiccation sensitivity, germination and chemical composition of *Q. ilex* acorns have been the subject of some study (Valero-Galván et al., 2011, 2021a; Pasquini et al., 2012; Joët et al., 2013; Romero-Rodríguez et al., 2014, 2015, 2018, 2019; Sghaier-Hammami et al., 2016, 2021; López-Hidalgo et al., 2021). Despite its significance to seed propagation and conservation, germination at the molecular level in recalcitrant seeds remains poorly understood. In this work, molecular events occurring during germination of seeds of the recalcitrant species *Q. ilex* and, specifically, in proteases and protease inhibitors (PIs), were examined with *in silico* and wet methods.

Maturation and germination in *Q. ilex* acorns have been the subjects of morphometric, physiological, transcriptomic, proteomic and metabolomic analyses (Valero-Galván et al., 2011, 2021b; Romero-Rodríguez et al., 2015, 2018, 2019; Sghaier-Hammami et al., 2016, 2021; Rey et al., 2019; Escandón et al., 2021a). Multiomic approaches have allowed differences in phytohormone, sugar and phenolic metabolism, proteases, ROS and storage proteins, and late embryogenesis proteins during seed germination to be detected (Romero-Rodríguez et al., 2018, 2019; Sghaier-Hammami et al., 2021). Also, regulation through post-translational modifications such as phosphorylation has been reported (Romero-Rodríguez et al., 2015). Romero-Rodríguez et al. (2019) observed accumulation of proteases and enzymes of amino acid metabolism in mature seeds. Their results support the hypothesis that mature nonorthodox *Q. ilex* seeds possess the machinery needed to rapidly resume metabolic activities and start germination.

Proteases are involved in almost all aspects of plant growth and development including germination, circadian rhythms, senescence, programmed cell death and responses to environmental changes (García-Lorenzo et al., 2006). Together with specific endogenous inhibitors that regulate their activities, proteases are in fact the main actors in effecting and regulating protein breakdown (Vaseva et al., 2011). During seed germination, much of the amino acid supply needed for emerging seedlings to grow comes from degradation of seed storage proteins. Proteases are not only important for storing hydrolysis proteins, but also responsible for other processes such as protein turnover, post-translational modifications, enzyme activation and inactivation, and plant defense (Schaller, 2004). Most proteolytic enzymes degrading seed storage proteins during seed germination are cysteine proteases, but others such as serine, aspartic and metalloproteases have also been reported (Tan-Wilson and Wilson, 2012). Protease activity is regulated at the transcriptional and translational levels but, most importantly, at the protein level. PIs are proteinaceous molecules that operate by regulating peptidase activity (Santamaría et al., 2014) and can have two different functions. Thus, they act as inhibitors of endogenous peptidases to regulate the activity of plant peptidases in order to avoid indiscriminate degradation when not needed (Martínez et al., 2012; Volpicella et al., 2012). Also, they regulate the activity of exogenous peptidases such as those used by various pests and pathogens to feed on and attack plants (Haq et al., 2004; Santamaría et al., 2012; Hörger and van der Hoorn, 2013).

Proteolytic enzymes have been classified according to catalytic mechanism, substrate specificity, cell locus, structure and function (Rawlings and Barrett, 1993; Van der Hoorn, 2008). The proteases and PIs in the MEROPS database^2^ contains a hierarchical classification where homologous sets of peptidases and protein inhibitors are grouped into families and clans (Rawlings et al., 2010). Complete sequence analysis of various genomes has shown that approximately 2% of the genetic information corresponds to proteases, which thus constitute one of the largest and best characterized functional groups of enzymes (Barrett et al., 1998). Plant genomes encode hundreds of proteases involved in a number of cellular processes; however, their regulation and subsequent actions are still poorly understood (van der Hoorn and Klemenčič, 2021).

Characterizing and comparing proteases and PIs in nonorthodox seeds such as those of *Q. ilex* is expected to improve our understanding of the role of these proteins in maturation and germination. In this work we conducted a comparative *in silico* analysis at the transcriptome level of *Q. ilex* and other *Quercus* species. Analyses were performed by using wet methods whereby protease and PI forms were examined at the proteomic level, and compared among *Q. ilex* organs. Changes in protease and PI profiles at the protein and activity levels were examined by using shotgun (LC–MSMS) proteomic analysis, and *in vitro* and *in gel* activity assays.

## Materials and Methods

### Comparative *in silico* Analysis of Protease and Protease Inhibitors in the Gene Product Databases for *Quercus* spp.

Proteases and PIs in the species-specific *Q. ilex* database (Guerrero-Sánchez et al., 2017, 2019, 2021) were validated and classified against the Merops (see Footnote 2), Gene Ontology^3^ and Panther^4^ databases. The transcriptome *Q. ilex* DB is deposited at NCBI GEO (Gene Expression Omnibus) repository with the accession GSE145009.^5^ In addition, the numbers of proteases and inhibitors found in the transcriptome of *Q. ilex* DB were compared with those of other *Quercus* spp. For this purpose, we used the transcriptome or gene product databases for *Q. suber* (Ramos et al., 2018),^6^ *Q. robur* (Plomión et al., 2018)^7^ and *Q. lobata* (Sork et al., 2016, 2022).^8^ The previous annotated databases were searched with the following key words: protease, proteinase, peptidase, proteasome and protease inhibitor.

### Plant Material and Experimental Germination Design

Mature acorns were harvested from a *Q. ilex* tree located at 38° 19′ 46″ N, 5° 33′ 15″ W in Aldea de Cuenca (Córdoba, southern Spain). Once in the lab, acorns were sterilized in a solution of commercial bleach (1%), washed with abundant tap water and dried with filter paper. Then, they were allowed to germinate in the dark for 4 weeks at a mean temperature of 22°C and (50 ± 10)% relative humidity according to Simova-Stoilova et al. (2015). Viable and nonviable seeds—the latter including seeds that failed to germinate within 4 weeks—from mature acorns at stage T1 and NV, respectively, as well as germinated seeds at stages T2–T4, were also collected for analysis (Romero-Rodríguez et al., 2018; Sghaier-Hammami et al., 2020; Supplementary Figure S1). After sampling, embryonic axes and cotyledons were separated and ground under liquid nitrogen in a mortar for storage at −70°C. Three replicates per stage and type of tissue (embryo axis or cotyledon), and one pool of 20 acorns per replicate, were used.

### Protein Extraction, Identification and Quantification by Gel-nLC-Orbitrap/MS Analysis

Two different protein extractions protocols were followed for shotgun protein profiling and protease activity analysis. With the first, an amount of 100 mg of embryo axes or cotyledons was extracted by using the TCA–acetone/phenol protocol (Wang et al., 2006; Maldonado et al., 2008). For activity assays, 100 mg of each type of tissue was extracted by following a slightly modified version of the protocol of Amaral et al. (2020). First, 600 μl of extraction buffer [50 mm Tris–HCl, pH 7.4, containing 10% (v/v) glycerol, 0.25% (v/v) Triton X-100, 1 mm dithiothreitol-DTT and 3% (w/v) polyvinylpolypyrrolidone (PVPP)] was added to ground tissue and mixed by inversion. After centrifugation at 17000 *g* at 4°C for 30 min, the supernatant was collected for protein and activity determinations. Proteins were quantified by following the Bradford protocol according to the manufacturer’s (Sigma-Aldrich) instructions.

Samples containing 70 μg of protein were subjected to sodium dodecyl sulfate–polyacrylamide gel electrophoresis (SDS-PAGE) on 12% acrylamide for protein cleaning according to Romero-Rodríguez et al. (2019). The only resulting band was excised and digested with trypsin and the peptides thus obtained were dissolved in 50 μl of a 4% acetonitrile/0.25% formic acid mixture for loading into an Ultimate 3,000 nano-LC-MS-UHPLC- Orbitrap Fusion instrument from Thermo Fisher Scientific (Waltham, MA, United States; Gómez-Gálvez et al., 2020). MS/MS data were processed with the software Proteome Discoverer v. 2.3, also from Thermo Fischer Scientific, and identified by using the SEQUEST algorithm against the species-specific database of *Q. ilex* developed from the transcriptome (Guerrero-Sánchez et al., 2017, 2019, 2021), using the setting reported by San-Eufrasio et al. (2021). Proteins were quantified in relative form from the peak areas for the precursor ions, using the three strongest peptide ion signals for each protein (Al Shweiki et al., 2017). Raw data were deposited in the ProteomeXchange Consortium *via* PRIDE (Perez-Riverol et al., 2018), using the identifier PXD032845. The following criteria were used to deem identifications confident: score ≥ 2, coverage ≥15% and at least two different peptides for each protein.

### *In vitro* and *in gel* Proteolytic Activity Assays

Proteolytic activity was assessed by following the protocol of Coêlho et al. (2016) in slightly modified form. For this purpose, we used a 1.5% (w/v) azocasein solution in protease extraction buffer [50 mM Tris–HCl, pH 7.4, containing 10% (v/v) glycerol and 0.25% (v/v) Triton X-100]. The enzyme extract solution was prepared at a total protein concentration of 1 mg/ml in extraction buffer. The reaction mixture contained identical amounts of enzyme extract and azocasein solution (125 μl each) and was incubated at 35°C for 6 h. The reaction was stopped by adding 700 μl of 5% trichloroacetic acid (TCA). Then, the mixture was allowed to stand at room temperature for 10 min and centrifuged at 2000 *g* for 10 min before the supernatant was collected and supplied with 0.5 N sodium hydroxide in a 1:1 ratio for absorbance measurements at 440 nm. A blank prepared by mixing 5% TCA with the substrate before adding the enzyme extract was also measured in parallel.

In gel protease activity was determined according to Heussen and Dowdle (1980). Protein extracts (40 μg per sample) were separated on 9% acrylamide–0.1% gelatin copolymerized gels. Samples were loaded with nondenaturing buffer [62.5 mm Tris–HCl containing 10% (v/v) glycerol and 0.001% (w/v) Bromophenol Blue]. Electrophoresis runs were performed at 4°C, using a voltage of 50 V until the proteins entered the resolving gel and then raising it to 80 V through the end. Then, gels were incubated under agitation at room temperature in 2.5% (v/v) Triton X-100 for 30 min, washed 3 times with distilled water and incubated overnight in proteolysis buffer (100 mm citrate buffer, Na_2_HPO_4_/citric acid pH 6.8, 4 mm DTT and 10 mM cysteine) under continuous shaking at 35°C. Proteolysis was stopped by transferring gels to a solution containing 0.1% (w/v) Coomassie Brilliant Blue R-250 (Neuhoff et al., 1988). Gel images were acquired with a calibrated GS-900 densitometer from Bio-Rad (Hercules, CA, United States). The effect of the PIs ethylenediaminetetraacetic acid (EDTA) and phenylmethylsulfonyl fluoride (PMSF) was evaluated by following the above-described protocol except that the sample was supplied with the corresponding inhibitor at a 100 mm concentration prior to use. Molecular weight of bands was calculated by mobility comparisons with protein standards markers (Thermo scientific Spectra Multicolor Broad Range). Protease activity bands were excised from the gels for shotgun analysis. Ten bands were cut from protease activity gels corresponded to the higher activity. Then, the bands were digested and analyzed by shotgun proteomic analysis such as described above.

### Statistical and Bioinformatic Analyses

Statistical analyses of shotgun and *in vitro* protease activity were performed with the core functions in the software R v. 3.6.2 (R Core Team, 2018) and the package pRocessomics v.1.8.^9^ Following Valledor et al. (2014), proteomic data were preprocessed with the Random Forest algorithm for assignation of missing values, using a threshold of 0.34 and a consistency-based criterion of 0.2. The abundance of each variable was normalized by following a sample centric approach. Preprocessed, filtered data were z-centered to ensure normality and homoscedasticity. Scaled and centered values (*z*-scores) were subjected to multivariate analysis, using the Manhattan distance method for heatmapping with the pheatmap library (Kolde, 2015). Venn diagrams were constructed and Principal Component Analysis (PCA) was done with pRocessomic and UpsetR v.1.4. (Lex et al., 2014). Univariate analyses (one-way ANOVA) and Tukey’s HSD *post-hoc* test were conducted on proteins and activities with *p* ≤ 0.05 and FDR (5%).

## Results

### *In silico* Analysis of Proteases and Protease Inhibitors in the *Quercus ilex* Transcriptome Database, and Comparison With Other *Quercus* spp.

Proteases and PIs in the species-specific *Q. ilex* transcriptome database (Guerrero-Sánchez et al., 2017, 2019, 2021) were subjected to *in silico* analysis (Supplementary Table S1). This database contains 45,815 transcripts 2,240 and 97 of which are annotated as proteases and protease inhibitors, respectively. These transcripts belong to the different protease families in MEROPS^10^ and were classified according to GO into biological process, molecular function and cellular component categories (Supplementary Table S1). Serine proteases and metalloproteases accounted for one-half of all proteases in the *Q. ilex* database, and degradation and metabolic proteins were highly represented. Also, one-half of the proteins in this group were predicted to be extracellular or membrane proteins. In addition, 50% of PIs were of the serine family, and 40% had defense and stress response functions.

Similarly, large numbers of proteases and PIs were previously found in other *Quercus* spp. Thus, 40,599 *Q. suber* transcripts have been listed, 974 being proteases and 17 PIs (Ramos et al., 2018).^11^ The *Q. lobata* database (Sork et al., 2022; see Footnote 8) contains 39,373 protein-coding genes with 721 corresponding to proteases and 44 to PIs. The figures for *Q. ilex* are similar to those for *Q. robur*, with 43,240 protein-coding genes (Plomión et al., 2018; see Footnote 7) 1815 of which are annotated as proteases and 173 as PIs.

### Shotgun Proteomic Analysis of Proteases and Protease Inhibitors in *Quercus ilex* Seeds: A Comparison of Tissues

A shotgun proteomics strategy was used to analyze the protein profile of seed cotyledons and embryo axes tissues from viable (T1 and T3) and nonviable (NV) seeds. In total, 3,512 proteoforms were identified in *Q. ilex* embryo axes and cotyledons. Editing and normalizing the data yielded a total of 1926 confident proteins (Supplementary Table S2a). PCA of the raw data (Supplementary Table S3; Supplementary Figure S2) revealed that the first two components accounted for 41% of total variability, PC1 mostly separating stages and PC2 tissues.

**Figure 1:**
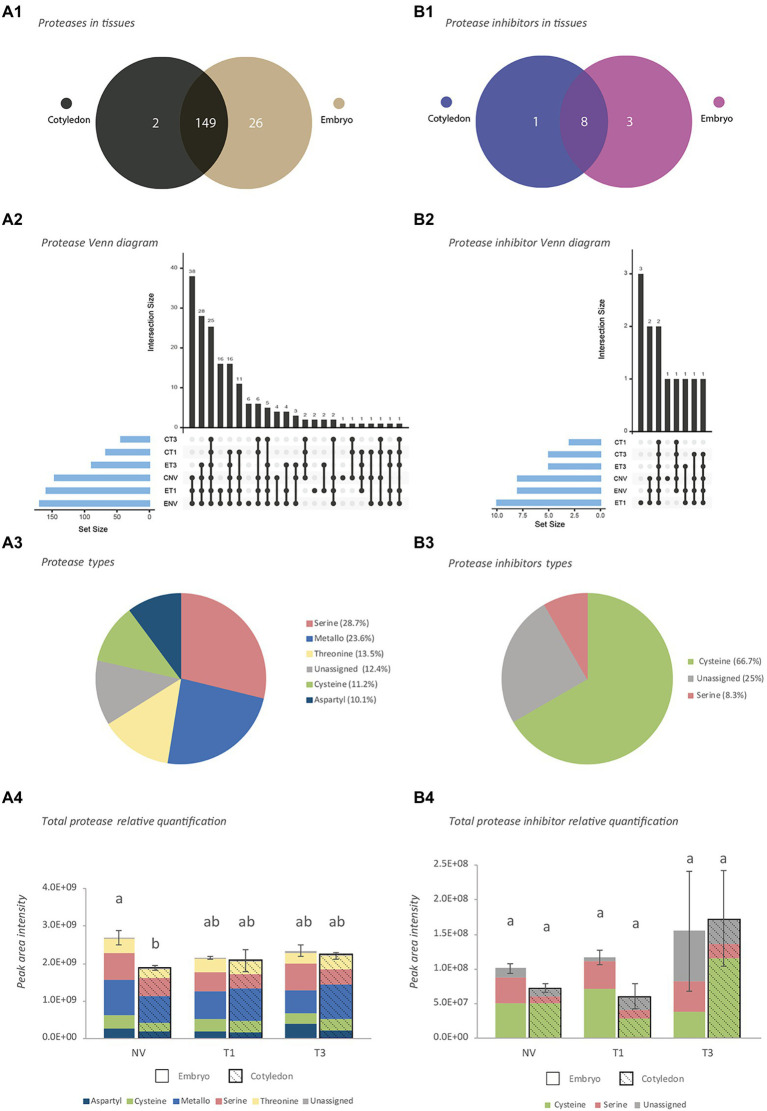
Characterization of proteases and protease inhibitors identified by shotgun analysis. **(A)** Proteases. **(B)** Protease inhibitors. 1: Venn diagram at tissue level, 2: Venn diagram at stage level, 3: Activity-type classification, 4: Total protein abundance based on combined peak areas. NV: Nongerminated acorns after 4 weeks of germination. T1: mature acorns prior to germination. T3: germinated acorns when root tip size reached 6.5 mm. The same letter indicates that there is no statistical difference between tissues or stages.

Proteins were then analyzed and classified in terms of protease activity as verified against the GO, Merops and Panther databases. This allowed a total of 177 proteases (Supplementary Table S2b) and 12 PIs (Supplementary Table S2c) to be identified. Most proteases (149) and PIs (8) were present in both embryo axes and cotyledon tissues (Figures 1A1,B1), 2 proteases and 1 PI being unique to cotyledons, and 26 proteases and 3 PIs to embryo axes.

In terms of germination stage, NV and T1 embryo axes, and NV cotyledons, contained the largest numbers of proteases and PIs (Figures 1A2,B2). These embryo axes and cotyledons shared 38 proteases and 2 PIs. More than one-half of all proteases identified belonged to the serine (28.7%), and metalloprotease (23.6%) families, followed by the threonine (13.5%), cysteine (11.2%) and aspartyl (10.1%) families (Figure 1A3). The remaining proteases (12.4%), which belonged largely to the 26S proteasome, could be assigned to no particular family. The vast majority of PIs (66.7%) were cysteine protease inhibitors, followed by an unassigned group (25%) and serine proteases (8.3%; Figure 1B3).

A plot of total protein abundance for the identified proteases and PIs based on their combined peak areas (Figures 1A4,B4) revealed differences that were significant in NV seeds only, where total proteases were better represented in embryo axes. Proteases were well represented by the metallo and serine families in both types of tissues at all stages (Figure 1A4). Although the most common PIs were from the cysteine family, the serine family was also well represented in embryo axes (Figure 1B4).

A comparison of the results of our shotgun proteomic analysis of *Q. ilex* seeds with those of previous studies (Sghaier-Hammami et al., 2020, 2021; Escandón et al., 2021b; Guerrero-Sánchez et al., 2021) allowed the identification of 400 proteases and 23 PIs differentially accumulating in various tissues (embryo axis, cotyledon, leaf and root) of *Q. ilex*. The largest number of them (354) was found in leaves, although a high number (129) were also present in all tissues. Several proteins were specifically detected depending on samples: 133 in leaves, 8 in a mix composed by acorn, leaf and roots, and 1 in acorns (Supplementary Table S2d). The best represented among them belonged to the serine and metalloprotease families, which together accounted for more than one-half of all identified proteins.

### Differential Proteases and Protease Inhibitors Between Embryo Axes and Cotyledons, and Seed Developmental Stages

**Figure 2:**
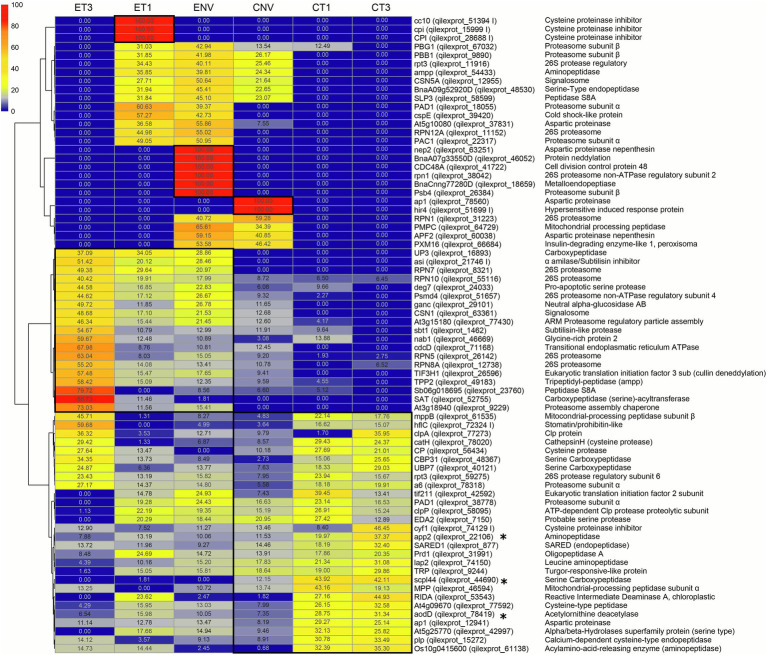
Proteases and protease inhibitors heatmap included in the top300 loadings of PCA and Venn specific tissue/stage protease. E, embryo axis. C, cotyledon. NV: Nongerminated acorns after 4 weeks of germination. T1: mature acorns prior to germination. T3: germinated acorns when root tip size reached 6.5 mm. ^*^ proteases present in both the first and second PC. Numbers in each cell represent the normalized abundance of the proteins found in each treatment. This abundance is reflected in the color scale shown.

Based on the PCA results, those proteases and PIs most markedly contributing to variability were selected for further investigation (Supplementary Table S3). For that we established a cut off at the 300 proteins contributing with higher loadings (absolute values) to the PC1 and PC2. A total of 55 proteases and 3 PIs were thus identified in this group, of which 3 proteases (*viz.*, carboxypeptidase scpl44, qilexprot_44690; Xaa-Pro aminopeptidase 2, qilexprot_22106; and acetylornithine deacetylase aodD, qilexprot_78419) were present in both components. A heatmap for the quantities of PCA-selected proteins that were differentially present in tissues or stages (Figure 2) exhibited two clusters. One included highly represented proteins in mainly ET1 and ENV; the other, encompassed two subclusters containing proteins that were more abundant in embryo axes (mainly in ET3) or cotyledons (mainly in CT1 and CT3). As can be seen from the heatmap, proteases accumulated more markedly in embryo axes than they did in cotyledons.

A number of RPN proteins (26S proteasome) fell in the highly represented group ET3, namely: qilexprot_8321, qilexprot_55116, qilexprot_26142, qilexprot_12738, and the 26S protease regulatory subunits qilexprot_51657, qilexprot_59275 and qilexprot_78318. Interestingly, 3 cysteine proteinase inhibitors (qilexprot_51394, qilexprot_15999, qilexprot_28688) were ET1-specific, and 8 proteases including the aspartic proteinases qilexprot_63251, qilexprot_78560 and qilexprot_60038 were NV-specific in both types of tissues.

T3 stage showed a marked increase of the proteases and PIs analyzed. Among them, 5 serine carboxypeptidases (*viz.*, qilexprot_44690 in CT3, qilexprot_16893 and qilexprot_52755 in ET3, and qilexprot_48367 and qilexprot_40121 in both CT3 and ET3) and 4 cysteine proteases (*viz.*, qilexprot_78020 and qilexprot_56434 in both tissues, and qilexprot_77592 and qilexprot_15272 in cotyledons). Embryo axes of the three stages differentially accumulated 2 signalosome (CSN) proteins (qilexprot_12955, qilexprot_63361) and 2 neddylation proteins (qilexprot_26596, qilexprot_46052).

### *In vitro* and *in gel* Protease Activity

**Figure 3:**
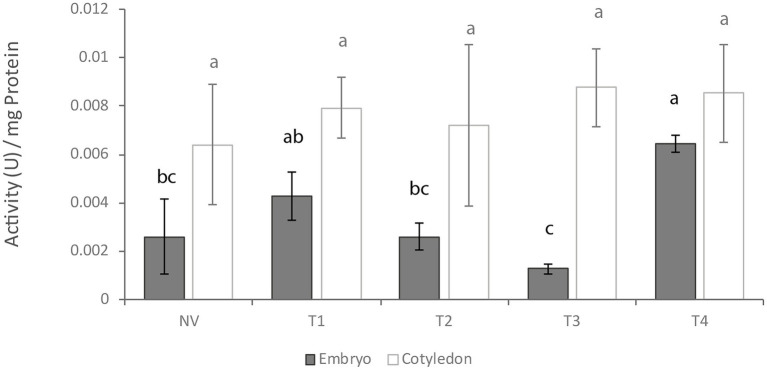
*In vitro* protease activity at different germination stages. NV: Nongerminated acorns after 4 weeks of germination. T1: mature acorns prior to germination. T2, T3, T4: Germinated acorns when root tips were visibly emerging from cotyledons (T2), their size reached 6.5 mm (T3) and it exceeded 20 mm (T4). Different letters denote significant differences between germination stages in each tissue.

*In vitro* protease activity as determined according to Heussen and Dowdle (1980) differed between embryo axes and cotyledons, being significant at T3 stage. Cotyledon tissues exhibited higher baseline activity at all germination stages except T4 (Figure 3). However, only embryo axis tissues differed throughout the germination process, with a decrease at T3 followed by an increase at T4.

**Figure 4:**
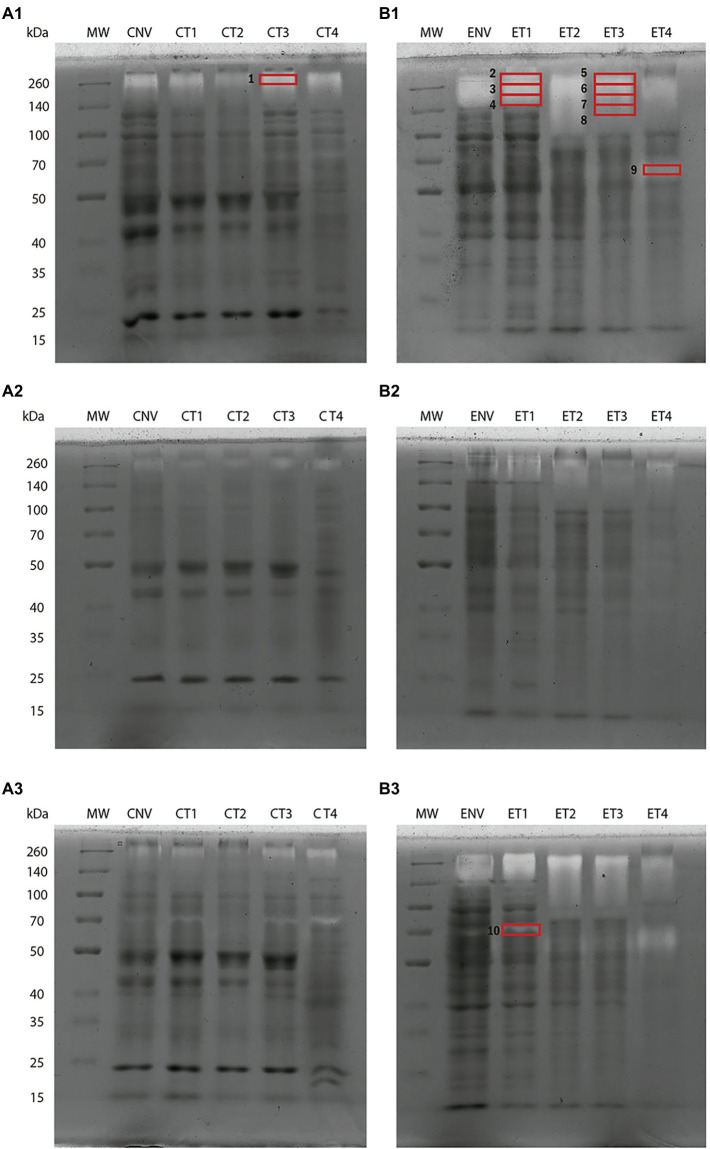
Protease activity in 9% polyacrylamide gel slabs containing gelatin at different germination stages. Cotyledon samples **(A)**. Embryo axis samples **(B)**: Gels containing no inhibitor (1); an inhibitor of serine protease activity (PMSF; 2); and one of metalloprotease activity (EDTA; 3). NV: Nongerminated acorns after 4 weeks of germination. T1: mature acorns prior to germination. T2, T3, T4: Germinated acorns when root tips were visibly emerging from cotyledons (T2), their size reached 6.5 mm (T3) and it exceeded 20 mm (T4). Ten bands were cut from protease activity gels for shotgun analysis (red boxes).

As can be seen from Figure 4, *in gel* activity profiles differed between cotyledons and embryo axes. Both types of tissue gave a band at *ca.* 260 kDa irrespective of developmental stages (Figures 4A1,B1). Such a band, however, was broader in embryo axes, where it ranged from 260 to 140 kDa at NV and T1, and from 260 to 100 kDa at T2, T3 and T4. A thin band was also seen at 70 kDa in cotyledons, and another at about 60 kDa in embryo axes, both of which increased at later stages.

Treating samples with the serine protease inhibitor PMSF (Figures 4A2,B2) considerably reduced band strength, which suggests the presence of abundant serine proteases in the extracts from both types of tissues. This finding is consistent with the number of serine proteases identified in the shotgun analyses. The metalloprotease inhibitor EDTA (Figures 4A3,B3) inhibited protease activity less markedly—so much so that the band of 70 kDa was even stronger in both embryo axes and cotyledons, possibly as a result of EDTA inhibiting certain PIs of the metalloprotease family.

Shotgun analysis of gel activity bands allowed the proteases and PIs potentially contributing to the greatest extent to some functions to be identified (Figure 4). A total of 224 proteases and 3 PIs were identified, mostly of serine, cysteine and metallo types. Band 1 (~260 kDa) was cut from cotyledons at T3 and allowed a leucine aminopeptidase (qilexprot_74150) to be identified with a high score. Bands 2–4 from embryo axes at T1 (260–140 kDa) revealed the presence of two cysteine protease inhibitors (qilexprot_28688 and qilexprot_15999) that were consistently represented in the three fractions. The serine peptidase qilexprot_24033 and the oligopeptidase qilexprot_31991 were also represented in them. The most salient proteins identified from bands 5–8 in T3 embryo axes (260–120 kDa) were the 26S proteasome regulatory subunit qilexprot_31223, clpA (qilexprot_77273), transitional endoplasmic reticulum ATPase (qilexprot_71168) and serine protease qilexprot_24033. Band 9 (~60 kDa) corresponded to T4 embryo axes, stage not examined in the whole shotgun analysis. Some proteins identified in embryo axes of T3 stage have been found in band 9. Such was the case with mitochondrial processing peptidase subunit beta (qilexprot_61535), transitional endoplasmic reticulum ATPase (qilexprot_71168), ubiquitin carboxyl-terminal hydrolase (qilexprot_40121), SAT1 (qilexprot_52755), serine protease (qilexprot_24033), ARM proteasome regulatory particle assembly (qilexprot_77430) and clpA (qilexprot_77273). Finally, band 10 (70 kDa), which was extracted from EDTA treated gel containing T1 embryo axis, allowed 5 aminopeptidases (qilexprot_74150, qilexprot_22106, qilexprot_49183, qilexprot_54433 and qilexprot_61138), 3 serine endopeptidases (qilexprot_48530, qilexprot_24033 and qilexprot_1,462) and 1 metalloprotease (qilexprot_31991) to be identified. The proteases and PIs identified with high scores (≥4) in gel activity bands are listed in Supplementary Tables S2b,c, respectively. Some proteases and PIs identified using the whole proteome were also identified in the bands from activity gels in the same experimental system.

## Discussion

Eukaryotes have a high number of protease-coding genes. More than 641 and 677 protease genes have been identified in the human and mouse genome, respectively (Bond, 2019). Numbers are even greater in plants, with 723 in *Arabidopsis* and 955 in *Populus*, for example (García-Lorenzo et al., 2006). The numbers for *Q. ilex* found here, and those previously reported for other *Quercus* species, are roughly similar if one considers post-transcriptional variants, the high level of heterozygosity in the genus *Quercus* and genome assembly errors (Sork et al., 2016, 2022; Plomión et al., 2018; Ramos et al., 2018). Thus, *Q. ilex* has 2,240 transcripts for proteases (Guerrero-Sánchez et al., 2017, 2019, 2021), *Q. suber* 974 (Ramos et al., 2018), *Q. lobata* 721 (Sork et al., 2022) and *Q. robur* 1815 (Plomión et al., 2018). The number of PI genes in these species is much smaller, with 97 transcripts in *Q. ilex* (this work), 17 in *Q. suber* (Ramos et al., 2018), 44 in *Q. lobata* (Sork et al., 2022) and 173 in *Q. robur* (Plomión et al., 2018). Also, the number of PIs in *Q. ilex*, 97, is much greater than those reported for other plant species such as tomato, with 55 (Fan et al., 2019).

Proteases in *Q. ilex* are complex in specificity, structure and catalytic properties, with representatives of the different families in MEROPS classification involved in various biological processes and belonging to the different molecular and cell locus groups in GO. The serine and metallo families are the best represented protease groups (especially those in the protein degradation, protein metabolic processes, and extracellular and membrane locus groups). As noted by Puente and López-Otín (2004), the views on proteases have changed since early reports from their being deemed nonspecific enzymes involved in proteolysis and protein catabolism to their enacting specific, limited selective cleavage of proteins, and regulating proteostasis and developmental processes ranging from growth and development to responses to environmental conditions. Seed germination is one of the biological processes where proteases play a key role; thus, they hydrolyze and mobilize reserve proteins (Martinez et al., 2019). Based on protein annotations in *Q. ilex* DB, 6% of the total proteases could be related to seed germination or embryo development, and 40% of the PIs with defense and stress response. The MEROPS database contains 83 family members of PIs, the serine family being the best represented in *Q. ilex* DB—mostly annotated with defense and response to stress roles. While PIs are present in all plant tissues, they are constitutive components of seeds and storage organs (McManus et al., 1999). Ever since they were assumed to be induced in response to pathogen and herbivore attacks (Ryan, 1990), PIs have been associated with defense responses. Interest in plant PIs has grown since the outburst of the COVID-2019 pandemic by effect of their potential against viral proteases (Hellinger and Gruber, 2019; Adhikari et al., 2021). Based on their abundance in some seeds, a twofold role can be envisaged, namely: protection against proteases from other organisms, and regulation of endogenous enzymes during mobilization of reserve proteins.

Out of the total 2,240 and 97 transcripts annotated as proteases and PIs, respectively, in the *Q. ilex* transcriptome, only 8% (177) and 12% (12) were detected by shotgun analysis of embryo axes and cotyledons here, 28 and 5, respectively, being tissue-specific. The differences between the two sets of data may be due to analytical or biological reasons such as differences in analytical potential between the two omic tools or in cell and temporal specific transcription and translation of genes. Including previously reported data for other organs increased the total number of proteoforms corresponding to proteases and PIs to 400 and 23, respectively, 129 being present in all organs (Sghaier-Hammami et al., 2020, 2021; Escandón et al., 2021b; Guerrero-Sánchez et al., 2021). The organ specific profile of proteases was reported by Hüynck et al. (2019). That for *Q. ilex* seeds is rich in serine and metallo proteases, and also in cysteine PIs, whereas that for cereal seeds is especially rich in proteases of the cysteine family, which mobilize and hydrolyze storage proteins (Martinez et al., 2019).

Comparing the results of the shotgun analyses among samples suggested that some of the proteins identified were also developmentally regulated. Thus, 11 proteases associated with mobilization of storage proteins were up-accumulated in T3 cotyledons and embryo axes, namely: 3 aminopeptidases (app2-qilexprot_22106, acylamino-acid-releasing enzyme-qilexprot_61138 and lap2-qilexprot_74150), 6 serine carboxypeptidases (scpl44-qilexprot_44690, EDA2-qilexprot_7150, CBP31-qilexprot_48367, UBP7-qilexprot_40121, UP3-qilexprot_16893 and SAT-qilexprot_52755) and 2 cysteine proteases (cathepsin H-qilexprot_78020 and CP protein-qilexprot_56434). Only after the function of a protein has been elucidated, can its role be interpreted in biological terms; in fact, transcriptomic and proteomic analyses by themselves do not suffice for this purpose since post-transcriptional and post-translational events rendering proteins inactive should always be considered. Enzymes should be assessed for activity. In this work, *in vitro* and in gel activity were assessed in extracts from embryo axes and cotyledons at a mature stage and upon germination. Gel activity tests provided few bands compared to the number of proteases identified by shotgun, revealing differences in the analytical potential of the used approaches and the complexity of the mechanisms regulating protein synthesis and activation. The differences in gel activity patterns, and those in shotgun analysis results, between samples confirmed that some identified proteins were tissue-specific or stage-specific.

Let us now discuss the organ and developmental pattern of some identified proteases and their putative biological role in terms of shotgun and gel activity data. Shotgun and protease activity analyses revealed more marked differences in embryo axes than there were in cotyledons. In gel activity tests, proteases spanned the 260–60 kDa *Mr* range in embryo axes, and the 260–70 kDa range in cotyledons. Nonspecific exo- and endopeptidases (*viz.*, amino and carboxyl isoforms) involved in storage protein mobilization were identified including leucine aminopeptidases and cathepsin H. A leucine aminopeptidase (LAP) was previously identified in mature, nongerminated *Q. ilex* seeds by Romero-Rodríguez et al. (2019). LAPs are highly conserved exopeptidases and among the most frequently used enzymes as gene markers in forest genetics (Rudin, 1976). LAP in *Picea abies* and other conifers exhibits high variability and tissue-unspecific activity in seeds, needles and pollen (Müller-Starck and Hüttermann, 1981). In this work, lap2 proteins were also abundant in band 1 from T3 cotyledons of gels activity. Also, cathepsin H-like protein was identified in viable *Q. ilex* seeds but not in nonviable seeds; as a result, this protein could be used as a putative marker of seed quality. Cathepsin H-like is an aleurain isolated from the aleurone of barley seeds (Rogers et al., 1985; Holwerda and Rogers, 1992). By using fluorescence activity-based probes on germinated *Arabidopsis* seeds, Lu et al. (2015) demonstrated dynamic activity in aleurain-like proteases, cathepsin B-like protein and vacuolar processing enzymes concomitantly with remobilization of seed storage proteins.

Some protein targeted proteases such as those that are ATP-dependent (Adam, 2007) were identified here and in previous studies (Balbuena et al., 2011; Romero-Rodríguez et al., 2019). Such proteases include members of the Clp family. For example, ClpA and ClpP are proteolytic subunits of the ATP-dependent Clp protease, which is found in the chloroplasts of higher plants, and plays essential roles in modulating the availability of short-lived regulatory proteins and in removing abnormal or damaged proteins (Shikanai et al., 2001). Besides its proteolytic role, ClpA functions as a chaperone independently of ClpP (Wickner et al., 1994). ATP-dependent ClpC proteases were specifically identified in germinated embryos of *Araucaria angustifolia*, indicating that this enzyme is synthesized during germination (Balbuena et al., 2011). Several Clp proteases were identified by shotgun in T3 embryo axes and cotyledons in this study (*viz.*, ClpA, qilexprot_77273; ClpP, qilexprot_58095; and ClpB, qilexprot_8964), as well as in the activity gels: band 1 (qilexprot_8964), bands 7 and 8 (qilexprot_8964, qilexprot_77273, qilexprot_50467, qilexprot_50), and band 9 (qilexprot_8964, qilexprot_77273). Overall, accumulation of these proteins at T3 indicates active mobilization of reserve proteins, some of which were present in mature (T1) fruits, albeit to a lesser extent. As previously was postulated by Romero-Rodríguez et al. (2019), in contrast to that occurs in orthodox seeds, in which all metabolic activity ceases in mature dry seeds, mature *Q. ilex* seeds have the machinery necessary for rapidly resuming metabolic activities and start the germination process.

Seed quality and viability can be assumed to depend on seed storage age and conditions, so assessing seed viability after sourcing may be useful. Despite great progress in seed biology, the sequence of cellular events that dictate whether seeds can germinate is poorly understood. Chaitanya et al. (2000) observed a gradual decline in total protein content due to corresponding increase in protease activity preceding loss of viability in *Shorea robusta* seeds after 6 days of harvest. In addition, substantially higher amounts of protease activity in embryonic axes than in the cotyledons might underline a special role played by embryonic axes in inducing protease activity in storage tissues (Chaitanya et al., 2000). In this work, the differential accumulation in tissues of proteins of the 26S proteasome and the Constitutive Photomorphogenesis 9 (COP9) signalosome (CSN) in embryo axes was striking (especially at T3). The protease complex encompassed by the 26S proteasome, and various types of E3 ligases ubiquitinating target proteins, are two key actors in the mechanism governing accumulation of regulatory proteins involved in phytohormone and light signaling pathways, and ultimately determining seed germination potential (Oracz and Stawska, 2016). CSN was originally identified in plants (Wei et al., 1994) and subsequently in all eukaryotic organisms. It is highly homologous to the lid sub-complex of the 26S proteasome, which is the major proteolysis machinery in eukaryotic cells, and involved in various cellular and developmental processes where it is believed to regulate ubiquitin proteasome-mediated protein degradation (Wei and Deng, 2003). CSN regulates the Cullin–Ring ubiquitin ligase (CRL) complex, which is the main class of E3 ligase complexes in eukaryotes (Lydeard et al., 2013; Teixeira and Reed, 2013). The existence of super-complexes consisting of CSN, the 26S proteasome and cullin-based Ub ligases has been suggested (Peng et al., 2003; Huang et al., 2005). CSN5 subunit catalyzes the hydrolysis of NEDD8 proteins from CRL, so it is responsible for CRL deneddylation acting as a deactivator (Cope and Deshaies, 2003; Wei and Deng, 2003). According to Franciosini et al. (2015), COP9 is deactivated during the maturation of *Arabidopsis thaliana* embryos and subsequently reactivated at germination.

Interestingly, two CSN proteins (CSN5A, qilexprot_12955; and CSN1, qilexprot_63361), a cullin deneddylation protein (qilexprot_26596) and a neddylation protein (qilexprot_46052) were differentially identified between *Q. ilex* embryo axes stages, with the neddylation protein exclusively found in ENV and the deneddylation protein highly represented in ET3. In addition to the above-described proteins, a number of others belonging to the 26S proteasome complex—6 up-represented at ET3, and 4 at ENV and ET1—were identified. Seven other proteins were identified as proteasome subunit alpha (4) and proteasome subunit beta (3) accumulating in embryo axes many of which were also identified in gel activity bands from T3 embryo axes. Although no categorical explanation for the differential accumulation of proteins from the COP9 signalosome and the 26S proteasome at different developmental stages can be put forward, a clear connection exists with embryonic axes development in germinated seeds.

This suggests that degradation *via* CNS/26S proteasome systems early during *Q. ilex* seed germination is crucial for adequate seedling development. Subtilisin-like endopeptidase (qilexprot_1,462) was also highly accumulated in T3 embryo axes. This protein performs specific functions in plant development and in signaling cascades. For example, subtilisins play a major role in cuticle development during embryogenesis (Van der Hoorn, 2008).

A few other proteins were tissue-specific. Such was the case with the cysteine PIs qilexprot_51394, qilexprot_15999 and qilexprot_28688, which were exclusively identified in ET1 seeds—and also consistently identified in gel activity bands from ET1 seeds. Proteinase inhibitors may play a role as reserve proteins in plants (Richardson, 1991; Hansen et al., 2007) and be involved in plant defense mechanisms. Cysteine PIs were previously isolated from seeds of the *Enterolobium contortisiliquum* tree targeting larval growth inhibition in the pest *Collasobruchus maculatus* (Nunes et al., 2021). Several studies have shown feeding insects with transgenic plants that express proteinase inhibitors to delay insect growth and development, and to cause starvation and death (Koiwa et al., 1998; Schuler et al., 1998; Mosolov et al., 2001). These proteins may thus not only play a defensive role against a potential attack by pathogens at a maturate stage but also act as inhibitors of endogenous peptidases to regulate their activity. Three other proteins were specifically identified in NV seeds (*viz.*, the aspartic proteinases nepenthesin-qilexprot_63251, qilexprot_78560 and nepenthesin-qilexprot_60038). Aspartic proteases are known to play major roles in storage protein processing, nitrogen remobilization, biotic and abiotic stress responses, and senescence and programmed cell death (PCD; Ge et al., 2005; reviewed in Bekalu et al., 2020a). Nepenthesin was initially reported in pitcher fluid from the carnivorous plant *Nepenthes* (Vines, 1901). Apart from carnivorous plants, nepenthesins have also been found in *A. thaliana* leaves, stems, seeds and pods (Takahashi et al., 2008) and in 24 tissues during the life cycle of *Oryza sativa* (Chen et al., 2009) suggesting ubiquitous occurrence and multiple functions. Overexpression of the nepenthesin-1 gene in the endosperm of barley grains significantly reduces infection and disease progression of Fusarium head blight disease, and also accumulation of toxins from the fungus (Bekalu et al., 2020a,b). Although it is difficult to ascertain whether these proteins are involved in defense or senescence processes, or both, their absence from viable seeds could be used as an indicator of nonviability. Also, the number of identified proteins related to proteasome: 26S proteasome regulatory proteins, proteasome subunit alpha, proteasome subunit beta and neddylation, highly accumulated in NV could be indicating a senescence process.

## Conclusion

Analyzing proteases and protease inhibitors (PIs) at different developmental stages of *Q. ilex* seeds by using a combination of shotgun proteomics and protease activity tests allowed the identification of a number of tissue- and stage-specific proteins in addition to others that were present in all systems. Comparing the omic and enzymatic activity results revealed differences in analytical potential between the two approaches but also useful complementariness. Accumulation of some proteases at the T3 germination stage such as the cathepsin H cysteine protease and Clp proteases among others, suggests active remobilization of reserve proteins; some proteases, however, were also active during maturation, which confirms the hypothesis that nonorthodox seeds such as *Q. ilex* acorns continue to be metabolically active at this stage. The large number of subunits of the CNS and 26S proteasome complex differentially identified in embryo axes of the several stages suggests that protein degradation *via* CNS/26S plays a major role early in germination, so it could be a useful indicator of seed viability. On the other hand, aspartic proteases such as nepenthesins were exclusively identified in NV seeds, so their presence could be used as indicator of nonviability. In addition, the specific accumulation of three cysteine protease inhibitors in mature embryo axes raises the possibility of their playing a protective role against potential attacks by pathogens during maturation. These results are important with a view to conserving recalcitrant native seeds as they reveal that some proteases and PIs can be useful as indicators of seed viability and quality, and also for biotechnological purposes.

## Data Availability Statement

The datasets presented in this study can be found in online repositories. The names of the repository/repositories and accession number(s) can be found in the article/Supplementary Material.

## Author Contributions

MAC and JJ-N: conceptualization, supervision, and funding acquisition. ME, EB, TH-L, and MAC: methodology development and experimental design. ME, VG-S, and MAC: bioinformatics and statistical analysis. MAC and ME: writing—original draft preparation. MAC, ME, M-DR, and JJ-N: writing—review and editing. All authors have read and agreed to the published version of the manuscript.

## Funding

This research was funded by the Spanish Ministry of Economy and Competitiveness in the framework of Projects BIO2015-64737-R and PID2019-109038RB-I00.

## Conflict of Interest

The authors declare that the research was conducted in the absence of any commercial or financial relationships that could be construed as a potential conflict of interest.

## Publisher’s Note

All claims expressed in this article are solely those of the authors and do not necessarily represent those of their affiliated organizations, or those of the publisher, the editors and the reviewers. Any product that may be evaluated in this article, or claim that may be made by its manufacturer, is not guaranteed or endorsed by the publisher.

## Footnotes

^2^ https://www.ebi.ac.uk/merops/

^3^ http://geneontology.org/

^4^ http://www.pantherdb.org/

^5^ https://www.ncbi.nlm.nih.gov/geo/query/acc.cgi?acc=GSE145009

^6^ https://corkoakdb.org/

^7^ https://www.oakgenome.fr/

^8^ https://valleyoak.ucla.edu/genomicresources/

^9^ https://github.com/Valledor/pRocessomics

^10^ https://www.ebi.ac.uk/merops/cgi-bin/family_index?type=P

^11^ https://corkoakdb.org/home

## Supplementary Material

Supplementary Figure S1 Germination stages. T1: mature acorns prior to germination. T2, T3, T4: Germinated acorns when root tips were visibly emerging from the cotyledons (T2), their size reached 6.5 mm (T3) and it exceeded 20 mm (T4). The bar corresponds to 1 cm.

Supplementary Figure S2 Classification of germination stages from a PCA plot of shotgun data. The PCA encompassed all proteins. E, embryo axis. C, cotyledon. NV: Nongerminated acorns after 4 weeks of germination. T1: mature acorns prior to germination. T3: germinated acorns when root tip size reached 6.5 mm.
