# Supplementary figures and images for: Identification of Proteases and Protease Inhibitors in Seeds of the Recalcitrant Forest Tree Species Quercus ilex
Source: Front Plant Sci. 2022 Jun 27;13:907042. doi: 10.3389/fpls.2022.907042 (PMC9271950; doi:10.3389/fpls.2022.907042)

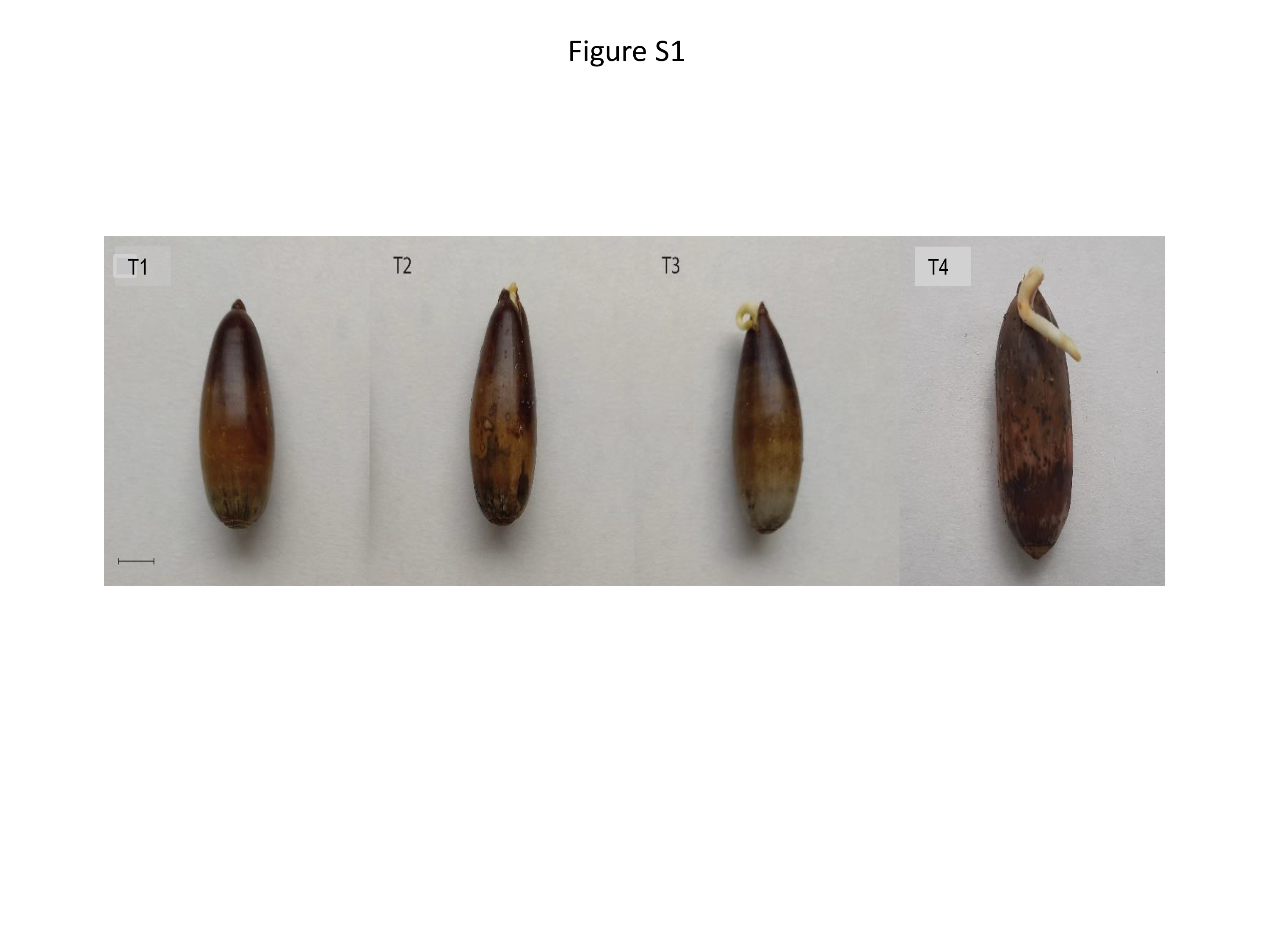

Supplement: Supplementary Figure S1 — Germination stages. T1: mature acorns prior to germination. T2, T3, T4: Germinated acorns when root tips were visibly emerging from the cotyledons (T2), their size reached 6.5 mm (T3) and it exceeded 20 mm (T4). The bar corresponds to 1 cm. [file Image_1.TIF]

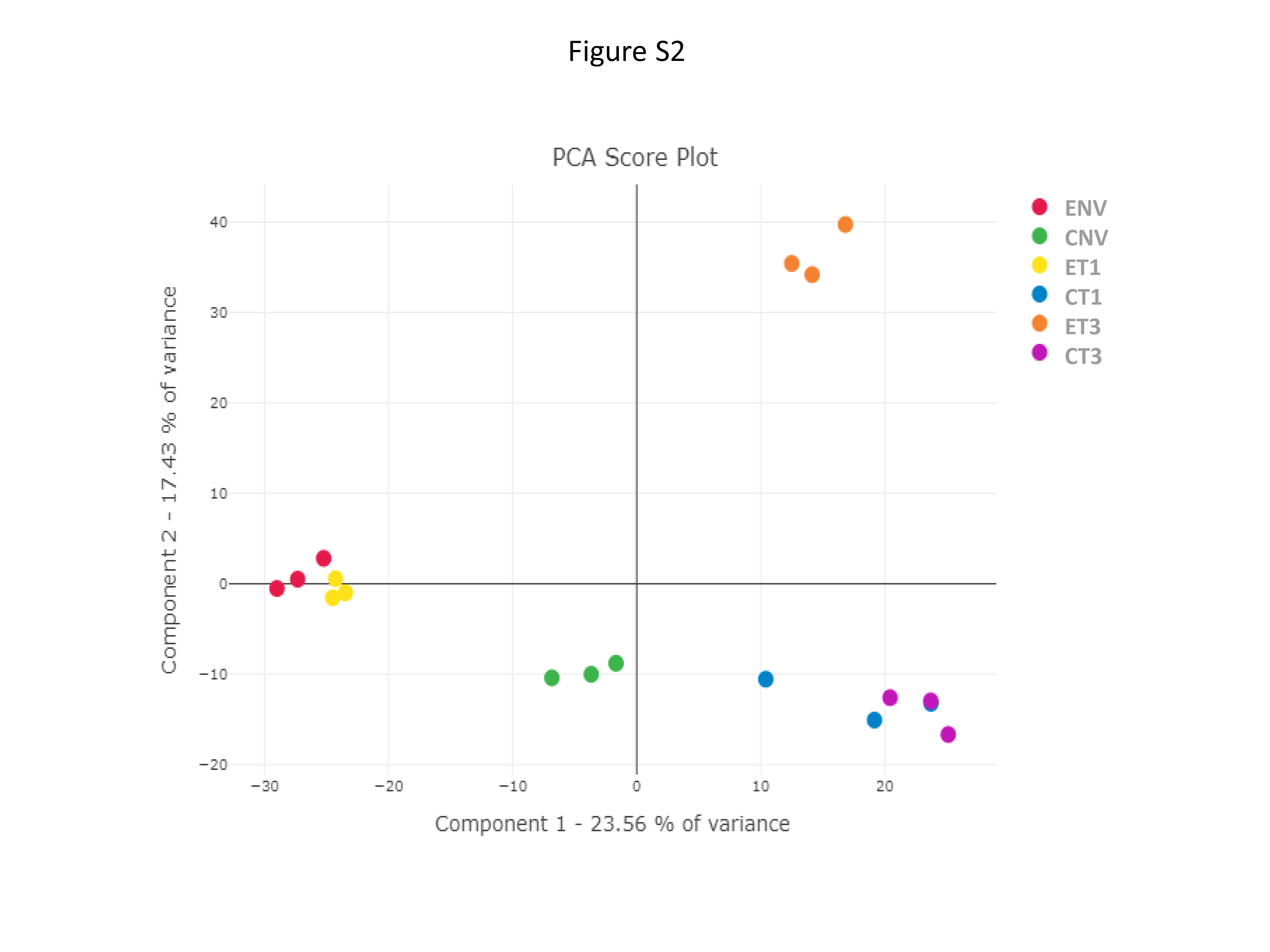

Supplement: Supplementary Figure S2 — Classification of germination stages from a PCA plot of shotgun data. The PCA encompassed all proteins. E, embryo axis. C, cotyledon. NV: Nongerminated acorns after 4 weeks of germination. T1: mature acorns prior to germination. T3: germinated acorns when root tip size reached 6.5 mm. [file Image_2.TIF]
